# Frequent Benign, Nontraumatic, Noninflammatory Causes of Low Back Pain in Adolescents: MRI Findings

**DOI:** 10.1155/2018/7638505

**Published:** 2018-02-08

**Authors:** Aikaterini Solomou, Pantelis Kraniotis, Aspasia Rigopoulou, Theodore Petsas

**Affiliations:** Department of Radiology, University Hospital of Patras, Patras, Greece

## Abstract

**Introduction:**

Low back pain (LBP) is common in children and adolescents. There are many factors that cause LBP, including structural disorders, degenerative changes, Scheuermann's disease, fractures, inflammation, and tumors. Magnetic Resonance Imaging is the gold standard for diagnosing spinal abnormalities and is mandatory when neurological symptoms exist. The study focuses on common MRI findings in adolescents with persistent LBP, without history of acute trauma or evidence of either inflammatory or rheumatic disease.

**Materials and Methods:**

Eleven adolescents were submitted to thoracic and/or lumbar spine MRI due to persistent LBP. The protocol consisted of T1 WI, T2 WI, and T2 WI with FS, in the axial, sagittal, and coronal plane.

**Results:**

MRI revealed structural abnormalities (scoliosis and kyphosis) in 4/11 (36.36%); disc abnormalities and endplate changes were found on 11/11 (100%). Typical Scheuermann's disease was found in 3/11 (27.27%). Endplate changes were severe in Scheuermann's patients and mild to moderate in the remaining 8/11 (72.72%). Kyphosis was in all cases secondary to Scheuermann's disease. Disk bulges and hernias were found in 8/11 (72.72%), all located in the lumbar spine.

**Conclusion:**

In adolescents with LBP, structural spinal disorders, degenerative changes, and Scheuermann's disease are commonly found on MRI; however, degenerative changes prevail.

## 1. Introduction

Low back pain is very common in children and adolescents. Underlying causes must be investigated by clinical and laboratory tests [1, 2].

MRI is the best method for the evaluation of the spine in the pediatric/adolescent population, due to its excellent tissue resolution, multiplanar capabilities, and lack of ionizing radiation. MRI is mandatory if neurological symptoms are present.

## 2. Material and Methods

We retrospectively reviewed 11 adolescent patients (6 male), age range 13–17 years, referred to MRI due to persistent LBP. The patients were submitted to MRI between April and November 2015. Patient's symptoms were dating back between 3 and 6 months. No patient was referred for typical clinical signs of radiculopathy or spinal cord compression. Patients with history of acute trauma, inflammatory, or rheumatic disease were excluded from the study. All patients were submitted to spine MRI with T1 WI, T2 WI, and T2 WI with FS, in the sagittal, axial, and coronal plane. The patients were submitted to MRI of the lumbar spine in 7/11 (63.63%) and thoracolumbar in 4/11 (36.36%) of the cases, according to the request by the referring clinician.

Patients were evaluated for the following:

(i) General structural spinal abnormalities like scoliosis or kyphosis.

(ii) Disc changes including narrowed disk space, bulges, and hernias, as well as presence of nerve root impingement. Nerve root impingement was graded as minor, moderate, or severe, with qualitative criteria by two radiologists, with consensus readings.

(iii) Vertebral changes comprising diffuse changes, or localized Schmorl's nodes and adjacent vertebral body marrow signal changes. The endplate changes were classified as minor, moderate, or severe, with qualitative criteria by two radiologists, with consensus readings.

(iv) Fulfillment of criteria for diagnosing Scheuermann's disease (i.e., vertebral wedging, with involvement of at least 3 adjacent vertebral bodies, anteroposterior elongation of vertebral bodies, endplate irregularity, and disc space narrowing) [3].

## 3. Results

Structural changes were found in 4/11 (36.36%) with scoliosis in 1/11 (9.09%) and kyphosis (secondary to Scheuermann's disease) in 3/11 (27.27%).

Disk changes were found in all 11/11 (100%) patients. Disk bulges/hernias were detected in 8/11 (72.72%). More specifically, there were disk bulges in 6/11 (54.54%) and hernias in 4/11 (36.36%). Patients showed narrowed disk spaces in 3/11 (27.27%) and Schmorl's nodes in 3/11 (27.27%).

Disk hernias were all located at the L5-S1 space, one of which with an extruding component. The anteroposterior dimension of the hernias was in the range 3–6 mm.

Nerve root impingement was absent in 5/11 (45.45%), mild in 1/11 (9.09%), moderate in 1/11 (9.09%), and severe in 2/11 (18.18%).

The criteria for diagnosing Scheuermann's disease were fulfilled by 3/11 (27.27%). All 3/3 (100%) patients showed severe endplate changes, narrowed disk spaces, and Schmorl's nodes.

**Figure 1:**
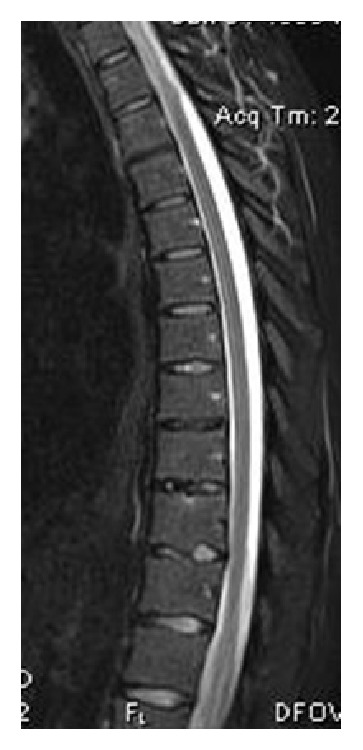
A 17-year-old male with Scheuermann's disease, depicted on the sagittal T2WI. There is multilevel involvement of the vertebral bodies, mainly in the lower thoracic spine. Minor anterior wedging, Schmorl's nodes, endplate irregularities, and disc space narrowing are present. Note the presence of kyphosis.

**Figure 2:**
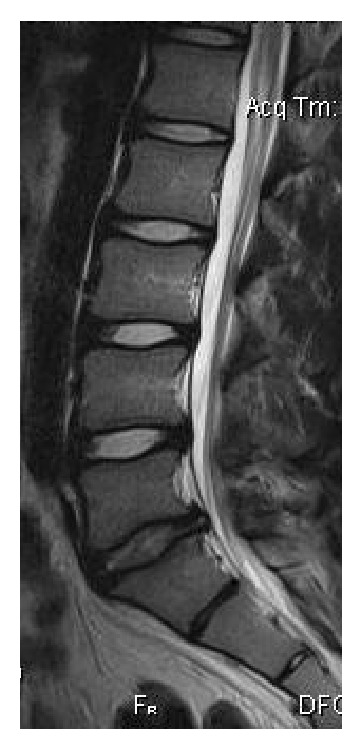
A 15-year-old male with disc bulging in the lower lumbar spine and a disc hernia at L5-S1 level, on sagittal T2WI.

**Figure 3:**
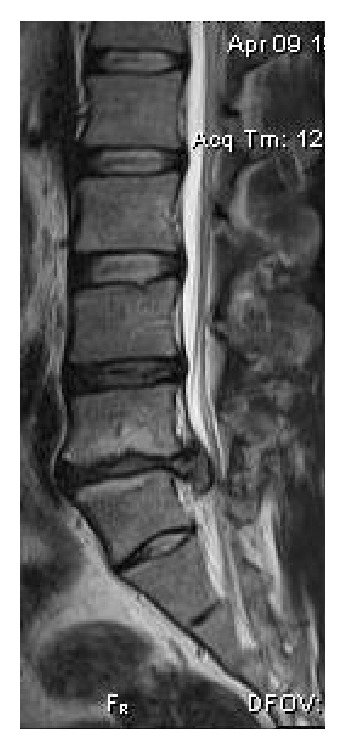
A 17-year-old male with an extensive descending disc hernia at L5-S1 level, with root impingement and severe degenerative endplate changes, on sagittal T2WI.

**Figure 4:**
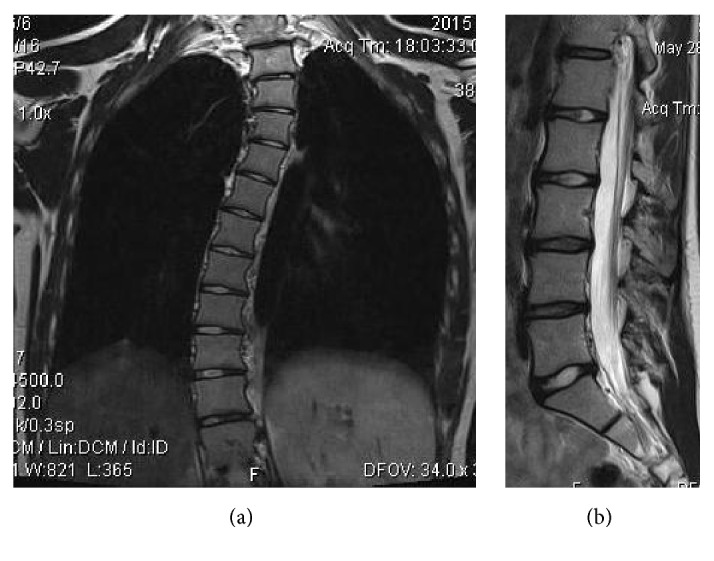
A 16-year-old female with thoracolumbar scoliosis. (a) Degree of scoliosis is appreciated on the coronal T2WI. (b) There is mild diffuse disc bulge and moderate degenerative disc changes at the L3-4 and L4-5 level, as well as moderate disk bulge at L5-S1 level, on the sagittal T2 WI.

Endplate changes were also detected in all 8/11 (72.72%) remaining cases, which were not fulfilling criteria for diagnosing Scheuermann's disease. These endplate changes were mild in 5/8 (62.50%) and moderate in 3/8 (37.50%). The results are summarized in Table 1. The main pathologic findings are shown in Figures 1–4.

## 4. Discussion

LBP is common in children and adolescents. Researches about children complaining for pain indicate that 1 in 20 will be suffering from LBP, at any time. According to a recent research, 19% of children between 11 and 14 years, who were free of symptoms at baseline, had a new episode of LBP in the next 12 months [4].

The one-month-period prevalence is 24% in children aged 11–14 years [5]. The one-year prevalence is 26% in children aged 12–17 years [6]. Actually the prevalence of back pain is lower among 7–10-year-old schoolchildren (ranging 1–6%) but increases with age, among 14–16-year-old adolescents (18%), with no gender difference. Chronic and recurrent low back pain is found more in girls (33%) than in boys (26%) and increases with age [7].

Many disorders may be responsible for LBP, such as structural disorders, degenerative changes, Scheuermann's disease, spinal or muscle trauma, infection, and tumors. MRI is considered the gold standard for the evaluation of spine, in children, not only due to its excellent tissue visualization and multiplanar capability but also because of the absence of radiation.

### 4.1. Structural Disorders and Scoliosis

Structural disorders and scoliosis are considered main causes for LBP. Scoliosis is common in children and is usually idiopathic, without associated structural defects. X-ray is the preferred method for the work-up and follow-up of scoliosis and may be sufficient pre- and postoperatively. The role of MRI is controversial. Some orthopedics prefer preoperative MRI in scoliosis patients, as it may be useful for diagnosing associated diseases, such as tumors, Chiari I malformation, intradural and extradural cysts, tethered cord, syringohydromyelia, and vertebral anomalies [8]. In our series only one female patient showed severe scoliosis, while kyphosis was secondary to Scheuermann's disease in all cases.

### 4.2. Degenerative Changes

Disc degeneration, called juvenile disc disorder or juvenile discogenic disorder (JDD), may develop at young ages. Many factors have been implicated, but repetitive microtrauma due to athletic activities and heavy back bags is the most frequently proposed cause [9]. When symptomatic, genetic factors should also be investigated. Nowadays, degenerative changes are more frequently detected in younger patients, due to the increased use of MRI. Advanced degenerative disease, such as severe disc narrowing may also be illustrated in X-rays, but MRI is considered the method of choice for the early detection of disk degenerative changes.

Interestingly, disc degeneration including bulging/protrusion or rupture of the annulus fibrosus can be observed in school-aged individuals, which is unusual. Neurological symptoms depend on the presence of nerve route compression [9].

In our study 8/11 (72.72%) of the young patients presented with degenerative disk disease, with male preponderance (6 male/2 female). These degenerative changes may be attributed to genetics, body habitus-obesity, smoking, environmental risks, and repetitive microtrauma [10, 11].

The most heavily affected disc was at the level of L5-S1 with associated hernias, whereas disc bulging was mainly affecting the lower lumbar spine, reflecting heavier load.

### 4.3. Scheuermann's Disease

Scheuermann's disease, a juvenile osteochondrosis of the spine, has a 0.4–8.3% incidence in the general population, being more frequent in male adolescents. This entity must be considered when investigating youngsters with LBP [12].

The cause of Scheuermann's disease is not proven, although mechanical compression during growth, acute disc injuries, hormonal variations, and genetic factors have been implicated. Scheuermann proposed that the kyphosis results from avascular necrosis of the ring apophysis of the vertebral body. Another contributing factor may be a biochemical abnormality of the collagen and matrix of the vertebral endplate cartilage [13].

Imaging investigation includes X-rays, CT, and MRI. MRI can reveal disc and endplate alterations and help diagnose the disease, according to relevant criteria [3].

Schmorl's nodes may be present in 30% of young patients. In our series they were present in all cases of Scheuermann's disease. Moreover all cases exhibited disk space narrowing and severe endplate changes.

Other frequent cases of LBP in children include acute trauma, infection, rheumatic disease, and tumors but these were beyond the scope of the study.

Injuries that occur due to repetitive trauma or untreated microtrauma are frequent, whereas acute traumatic fractures and severe injuries are less common [14].

Spinal infections, like vertebral osteomyelitis and discitis, are rare in children. Vertebral osteomyelitis accounts only for 2–4% of all cases of LBP [15] and is associated with fever and positive laboratory tests.

Primary osseous neoplasms of the lumbar spine are not common. The most frequent are Ewing sarcoma, benign osteoblastoma, aneurysmal bone cyst, osteoid osteoma, and primary lymphoma as well as spinal cord tumors such as ependymoma [14].

Study limitations may include the retrospective character and the relatively small series. Imaging of either thoracolumbar spine or only lumbar or thoracic spine alone, according to clinical referral, may also pose a bias.

## 5. Conclusions

In adolescents, complaining for persistent LBP, without history of either acute trauma or fever/positive lab tests for inflammation/rheumatic disease, MRI may commonly reveal degenerative disc changes and vertebral endplate changes. Disc changes are more frequent in the lumbar spine, with hernias being more frequent at the L5-S1 level. Scheuermann's disease is the second more frequent cause of LBP. Spine MRI is useful in the diagnostic work-up in adolescents with persistent LBP and may depict a variety of common underlying diseases, like structural disorders, degenerative changes, and Scheuermann's disease.

## Figures and Tables

**Table 1:** Age, gender, MRI findings, diagnosis.

	Gender	Age	MRI	Disc changes	Nerve root impingement	Endplate changes	Final diagnosis
(1)	Male	17	Lumbar	Disc bulges (L3-S1)	-	++	Degenerative changes
(2)	Female	13	Thoracic lumbar	Disc bulges (L3-S1)	-	++	Scoliosis/degenerative changes
(3)	Male	16	Lumbar	Disc bulges (L3-S1)	+++	+	Degenerative changes
(4)	Male	17	Lumbar	Disc bulges (L2–5) Disc hernia (L5-S1)	+++	++	Degenerative changes
(5)	Male	16	Lumbar	Disc bulges (L2–4) Disc hernia (L5-S1)	+	+	Degenerative changes
(6)	Male	17	Thoracic lumbar	Narrowing of disk space Schmorl's nodes	-	+++	Scheuermann's
(7)	Female	17	Thoracic lumbar	Narrowing of disk space Schmorl's nodes	-	+++	Scheuermann's
(8)	Female	14	Lumbar	Disc hernia (L5-S1)	++	+	Degenerative changes
(9)	Female	15	Thoracic lumbar	Narrowing of disk space Schmorl's nodes	-	+++	Scheuermann's
(10)	Male	16	Lumbar	Disc hernia (L5-S1)	+	+	Degenerative changes
(11)	Female	17	Lumbar	Disc bulges (L3–5)	+	+	Degenerative changes

(-) no pressure, (+) minor pressure/changes, (++) moderate pressure/changes, (+++) severe pressure/changes.

## Abbreviations

LBP: Low back pain
CT: Computed tomography
MRI: Magnetic Resonance Imaging
FS: Fat suppression.
